# Review of the Application of Modern Cytogenetic Methods (FISH/GISH) to the Study of Reticulation (Polyploidy/Hybridisation)

**DOI:** 10.3390/genes1010166

**Published:** 2010-07-02

**Authors:** Michael Chester, Andrew R. Leitch, Pamela S. Soltis, Douglas E. Soltis

**Affiliations:** 1 Department of Biology, University of Florida, Gainesville, Florida 32611, USA; E-Mails: mchester@ufl.edu (M.C.); dsoltis@ufl.edu (D.E.S.); 2 School of Biological and Chemical Sciences, Queen Mary, University of London, UK; 3 Florida Museum of Natural History, University of Florida, Gainesville, Florida 32611, USA; E-Mail: psoltis@flmnh.ufl.edu

**Keywords:** cytogenetics, ISH, polyploidy

## Abstract

The convergence of distinct lineages upon interspecific hybridisation, including when accompanied by increases in ploidy (allopolyploidy), is a driving force in the origin of many plant species. In plant breeding too, both interspecific hybridisation and allopolyploidy are important because they facilitate introgression of alien DNA into breeding lines enabling the introduction of novel characters. Here we review how fluorescence *in situ* hybridisation (FISH) and genomic *in situ* hybridisation (GISH) have been applied to: 1) studies of interspecific hybridisation and polyploidy in nature, 2) analyses of phylogenetic relationships between species, 3) genetic mapping and 4) analysis of plant breeding materials. We also review how FISH is poised to take advantage of next-generation sequencing (NGS) technologies, helping the rapid characterisation of the repetitive fractions of a genome in natural populations and agricultural plants.

## 1. Introduction 

**Figure 1:**
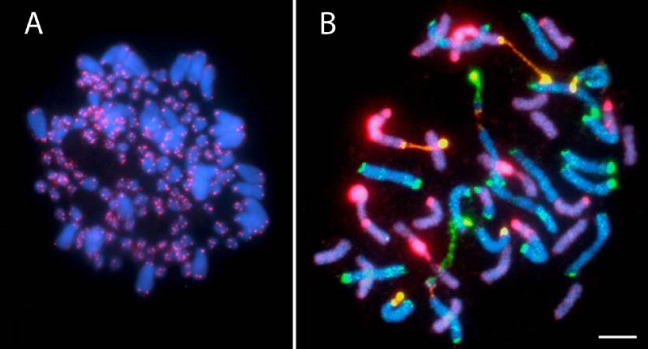
ISH to root tip metaphase spreads **(A)** FISH to pentaploid *Agave fourcroydes* (2*n* = 5*x* = 150) probed with telomere repeat sequences (TTAGGG)n (biotin labelled probe Cy3 signal, pink), chromosomes counterstained with DAPI (blue). **(B)** GISH to a partial metaphase spread of a *Nicotiana sylvestris* (2*n* = 2*x* = 24) x *N. tomentosiformis* (2*n* = 2*x* = 24) allotetraploid, which has a chromosome complement of 2*n* = 4*x* = 48 (see Kostoff hybrid [2]). The spread is probed with total genomic DNA from *N. sylvestris* (digoxigenin-labelled probe, FITC signal, green) and from *N. tomentosiformis* (biotin-labelled probe, Cy3 signal, pink), chromosomes are counterstained with DAPI (blue). Depending on the mixture and intensity of the fluorophores other colours are generated. Chromosomes of N. sylvestris origin are shown as cyan and green depending on the strength of the FITC/DAPI signal, while those of *N. tomentosiformis* origin are violet and magenta depending on the strength of the Cy3/DAPI signal. Areas of yellow chromatin represent 35S ribosomal DNA (rDNA) loci that are hybridised with genomic DNA from both parents. Scale bar: 10 µm.

Classical cytological studies using chromatin staining still contribute much to the present appreciation of chromosomal diversity in wild species. This approach allows the detection of gross karyotypic alterations such as changes in chromosome number and morphology and gives an overview of chromosome behaviour in mitosis and meiosis. However, many recent cytogenetic studies have used FISH to map single cloned or PCR-amplified sequences to chromosomes (Figure 1A) or GISH with total genomic DNA probes to identify the parental origin of chromatin in hybrids and allopolyploids (Figure 1B). FISH and GISH together have shed much light on many biological phenomena. Of particular interest to this review are their roles in improving our understanding of interspecific hybridisation and polyploidy, phylogenetic relationships, genetic mapping and plant breeding. These methods also have a role in clarifying patterns of chromatin folding, interphase nuclear organisation and chromatin distribution in different cells of the cell cycle and in development [cf. 1].

### Applications of ISH

Since GISH was first demonstrated in synthetic *Hordeum chilense* x *Secale africanum* [3] and *Triticum aestivum* (wheat) x *S. cereale* (rye) [4] it has been used extensively to track the artificial introgression of chromatin in wide crosses [5,6,7,8]. GISH has also been used to inform on the genome constitution of natural hybrids, sometimes in combination with FISH to identify the parental origin of specific loci. Bennett *et al.* [9] first used GISH for the purpose of confirming natural hybridisation in the allotetraploid *Milium montianum*. The results predicted an extinct or undiscovered *Milium* parent of *M. montianum*, demonstrating the method’s potential for paleoreconstructions of ancestral or extinct genomes. One question raised by the authors was whether GISH could provide a quantitative measure of chromosome divergence. GISH with a range of genomic DNAs from different species to metaphase spreads of a *Silene* hybrid** showed that the intensity of fluorescence varied quantitatively based on the relatedness of the species, as determined by divergence of ITS1-5.8S-ITS2 sequences [10].

ISH has been applied to allopolyploids identified via morphology, chromosome counts, ecology and other data both to investigate their parentage further and to evaluate chromosomal variation that has arisen since polyploid formation. ISH can provide further evidence for a hypothesised allopolyploid origin and reveal genomic changes, such as intergenomic translocations; examples include *Nicotiana* [11], *Lepidium* [12]*, Primula* [13] and *Oryza* [14]. Sequence and karyotype divergence has been examined using ISH in allopolyploid species in a phylogenetic context; examples include *Paeonia* [15], *Aloe* [16],* Clivia* [17], *Nicotiana* [18,19] and *Pinus* [20].

A powerful application of FISH in the genomic era is the physical mapping of eukaryotic genomes [21]. FISH can anchor BAC contigs to specific chromosome arms, making it possible to construct a comprehensive physical map [22,23,24,25,26]. BAC-FISH enabled the designation of six linkage groups to their corresponding chromosomes in cotton (*Gossypium hirsutum*, 2*n* = 4*x* = 52), and cross-hybridisation of BACs to two pairs of loci identified homeologous regions/chromosomes [27,28]. During the assembly of the *Carica papaya* genome, BAC-FISH was used to assign two linkage groups to one of the chromosomes [29]. Using elegant ISH strategies to paint entire chromosomes with BACs, translocations and rearrangements have been described in Brassicaceae, enabling the prediction of ancestral karyotypes [30,31]. This method has provided additional evidence for a hypothesised whole-genome triplication in the common ancestor of a clade that includes species of *Brassica* and *Sinapis* (tribe Brassiceae) [32,33,34].

## 2. Recently Formed Allopolyploid Species

**Figure 2:**
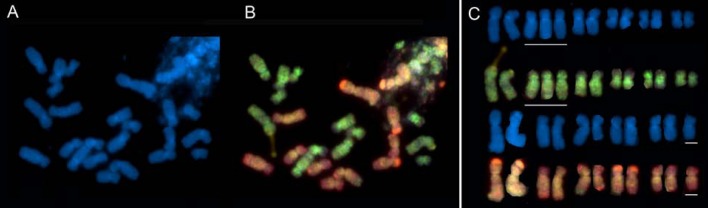
GISH to root tip metaphase chromosomes of a wild accession of *Tragopogon mirus* 2*n* = 4*x* = 24 -1 +1. **(A)** DAPI stained chromosomes. **(B)** Chromosomes hybridised with genomic DNA identifying subgenomes originating from either *T. dubius* (green) or *T. porrifolius* (red). **(C)** Karyotype with DAPI-stained chromosomes (from A) and genomic DNA hybridised chromosomes (from B) originating from *T. dubius* (top two rows) and *T. porrifolius* (lower two rows), respectively. White lines indicate trisomic and monosomic chromosomes, respectively.

**Figure 3:**
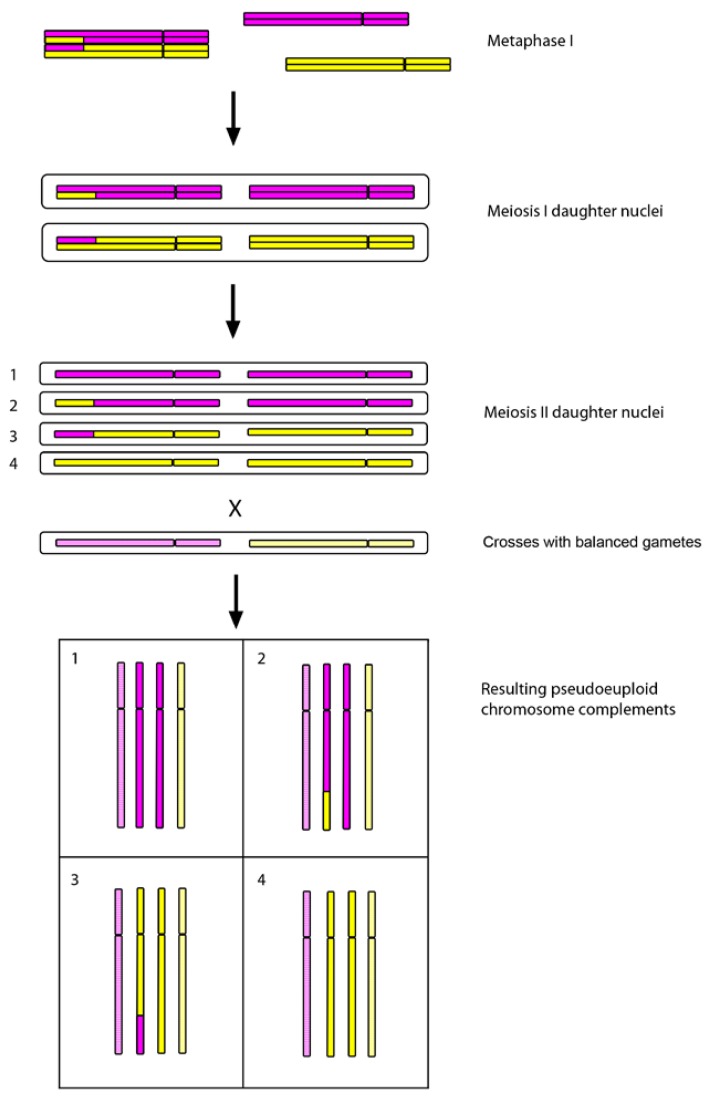
A model for pseudoeuploidy involving homeologous chromosomes. Homeologous chromosomes are labelled yellow or purple. An aberrant bivalent is formed between homeologous chromosomes, and a single crossover results in a homeologous translocation; the two remaining homeologues remain unpaired (univalents). Note that if the translocation had already occurred, this might lead to aberrant pairing. Due to missegregation, daughter cells of the 1st meiotic division receive two homologous chromosomes. The resulting gametes from the 2nd meiotic division have the correct numerical complement but are unbalanced. Plants arising from a cross between gametes 1-4 result in the corresponding complements (chromosomes shown in an unreplicated state). Note that other aberrant pairing configurations such as two homeologous bivalents or a quadrivalent may also result in homeologues segregating to the same daughter nuclei in the 1st meiotic division. Such aberrant meioses were observed in 1st-generation synthetic *Brassica napus* lines; monosomic/trisomic plants for chromosome 1 were generated at a frequency of ~5% following backcrossing to natural *B. napus*[41]. *Tragopogon* allotetraploids in the wild were found to exhibit reciprocal aneuploidy for homologous or homeologous chromosomes [40].

FISH and GISH have made a contribution to our understanding of early evolution (within the last 150 years) of recently formed polyploid species of *Tragopogon* and *Spartina*. Two allotetraploid species of *Tragopogon* arose in the early 1900s in North America, following the introduction of three diploid (2*n* = 2*x* = 12) species from Europe [35]. The polyploids (2*n* = 4*x* = 24) share one parent, *T. dubius*, which with *T. pratensis* gave rise to *T. miscellus* and with *T. porrifolius* gave rise to *T. mirus* [35]. Molecular studies of populations in the states of Washington and Idaho, USA, have identified many independent origins; essentially each population appears to represent a separate origin [36,37,38]. Furthermore, gene flow between populations appears to be absent or extremely rare [36], and initial experimental crosses between reciprocally formed *T. miscellus* populations failed, suggesting possible chromosomal incompatibilities [39]. Analyses of the polyploids using GISH revealed that in several numerically (2*n* = 24) euploid plants there was an uneven parental contribution, resulting in plants with karyotypes of 2*n* = 24 -1 +1 or 2*n* = 24 -2 +2. These plants were reciprocally aneuploid for the same or different homeologous chromosomes [40] (an example of the latter is shown in Figure 2). One way in which a pseudoeuploid chromosome complement could arise is through homeologous pairing and recombination, as was observed in the pollen mother cells of resynthesised *Brassica napus* (oil seed rape) [41], an example of which is shown in Figure 3. Such homeologous associations, if occurring over multiple generations, could explain some of the gene losses identified by homeoSNP-based studies [42,43,44,45,46].

The genus *Spartina* is the other example where GISH has played a role in our understanding of early allopolyploid evolution in nature. *Spartina anglica* (2*n* = 12*x* = 120, 122, 124) arose at Hythe in Southampton Bay, UK, following genome doubling of the sterile homoploid hybrid *S.* x* townsendii* (2*n =* 6*x* = 60, 62) in the late 1900s [47]. *Spartina anglica* has since colonised salt marsh and estuarine habitats around the world through human introductions and aggressive colonisation [48,49]. It is not known for certain if *S. anglica* arose from a single hybridisation event or multiple times, as the limited genetic variation that does exist can be explained by multiple origins or genomic restructuring following polyploid formation [50,51]. Using total genomic DNA probes of *S. alterniflora* (AA)** and *S. maritima* (MM), where each A or M represents three basic genomes of *x* = 10, Renny-Byfield *et al*. [52] were able to determine the parental origin of chromosomes in polyploid material. One of the individuals analysed was found to be a dodecaploid (12*x*, AAMM) as expected, while the other was a nonaploid (9*x*, AAM). Ploidy estimation by flow cytometry of individuals collected at the site showed that nonaploids were present at frequencies comparable to *S. anglica* and *S. maritima*. The data suggest that nonaploids may be arising at the site frequently; it is possible that these allow gene flow between 6*x* and 12*x* species [52].

## 3. Autopolyploidy, Polyploid Species Complexes and Reticulate Evolution

Some species have a complex range of chromosome numbers; for example, *Agrostis stolonifera* (Poaceae) has a polyploid series based on *n* = 7, with chromosome numbers of 14, 21, 28, 35, 42, 49, 56, 63 and 70, while *Cardamine pratensis* (Brassicaceae) has a series based on *n* = 8 with aneuploidy occurring at several of the ploidal levels (chromosome numbers are 16, 24, 28, 30, 32-38, 40-46, 48, 52-64, 67-96) [53]. Aneuploid gametes are produced by odd-ploidy plants usually in excess of euploid gametes [54], due to errors at meiosis such as unequal segregation and non-disjunction [55,56,57,58,58]. Furthermore, as exemplified by the *C. pratensis* series above, plants of higher ploidal levels tend to tolerate a greater degree of aneuploidy [59,60,61].

Polyploid series arising through autopolyploidy (chromosome set multiplication without interspecific hybridisation) or allopolyploidy have been difficult to distinguish when progenitors share the same basic chromosome number [62]. *Brachypodium distachyon* includes polyploids, with individuals being 2*n* = 10, 20 or 30. This chromosome series was investigated by Hasterok *et al*. [63], who examined karyotypes with FISH and GISH. The data show that a 2*n* = 30 individual was not an autopolyploid as thought, but an allopolyploid derived from at least two progenitors similar to *B. distachyon* and *B. sylvaticum*, contributing 10 and 20 chromosomes, respectively.

**Figure 4:**
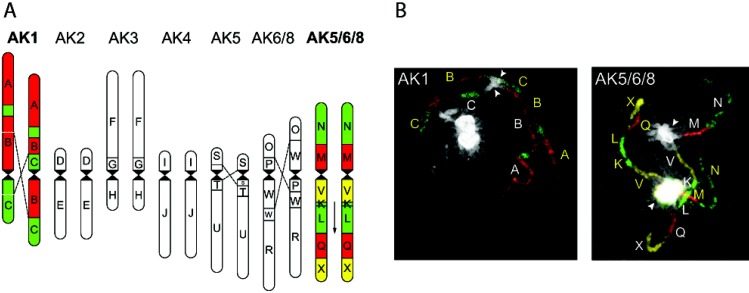
Idiogram and corresponding FISH to pachytene spreads using *Arabidopsis thaliana* genomic BACs to the autotetraploid *Goldbachia laevigata* (2*n* = 4*x* = 28) (Brassicaceae; Calepineae). **(A)** Chromosomes are annotated corresponding to the ancestral karyotype (AK) for crucifers [31,64]. Lines between chromosomes indicate the boundaries of pericentric inversions distinguishing homologous chromosomes, an arrow indicates an inversion relative to the crucifer AK. **(B)** FISH to two pachytene bivalents of AK1 and AK5/6/8 chromosomes hybridised with pooled BACs are shown pseudocoloured as red, yellow and green. Yellow and white letters distinguish each bivalent.

The homologous genomes of two putative autotetraploid species have been analysed using a BAC-FISH approach. Mandakova and Lysak [30] found no major rearrangements between the subgenomes of *Calepina irregularis* (2*n* = 4*x* = 28),****whereas *Golbachia laevigata* (2*n* = 4*x* = 28)****showed three alterations to colinearity between subgenomes (Figure 4). In the case of *G. laevigata* it may be that subgenome divergence occurred following autopolyploidisation or alternatively prior to the hybridisation event, which would suggest a possible allopolyploid origin [30]. 

Given sufficient genetic divergence, GISH can discriminate the parental origin of genomic DNA in hybrids, providing a powerful method to resolve patterns of reticulation in a species complex. An excellent example of this was described in the *Boechera* [*Arabis*]* holboellii* complex, which comprises *B. holboellii* (with variable chromosome numbers arising through polyploidy and aneuploidy), *B. stricta* (typically diploid) and their putative hybrid, *B. divaricarpa*, which probably arose through multiple hybridisation events [65,66]. Frequently, crosses between allotetraploids and their diploid parents result in a triploid block because the offspring produced are inviable or the endosperm fails to develop [67,68]. GISH identified some accessions of *B. divaricarpa* with unequal parental chromosome contributions. The results are indicative of crosses between differing ploidal levels and involving gametes generated from aberrant meiotic recombination and segregation [69]. Similarly, in “*B. holboellii*”, GISH revealed several chromosomes originating from *B. stricta*, *i.e.*, 4, 10 or 11 in the case of 2*n* = 15 individuals. Collectively, these data reveal much reticulate evolution and introgression of DNA in species of this complex.

**Figure 5:**
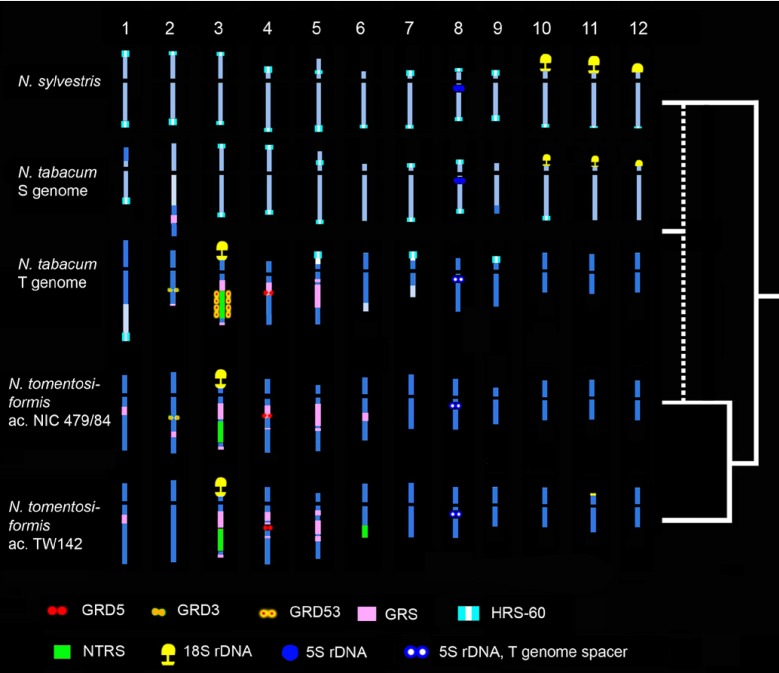
Idiograms for the subgenomes of *Nicotiana tabacum* and the extant species representing its hypothesised parental lineages. The positions of repetitive DNA clusters used for predicting genomic relationships are shown (for details see [73]). The phylogenetic scheme on the right indicates parental species divergence and the hybridisation event (indicated by dotted line) involving a specific lineage of *N. tomentosiformis*.

The presence or absence of repetitive sequences can be used to distinguish genetic lineages [70]. Within the genus *Nicotiana*,** several integrations of geminiviral related DNA (GRD) were found as distinct, tandemly repeated clusters, making them ideal FISH markers [71,72]. In section *Tomentosae*, mapping with GRD and other repeats enabled a phylogram to be constructed based on the presence or absence of homologous clusters [72]. Due to variation in the occurrence of a GRD3 cluster in the *N. tomentosiformis* subgenome of *N. tabacum* (tobacco),** a particular *N. tomentosiformis* lineage could be identified as a likely parent [73]. Furthermore, sequence analysis of the GRD sequences indicates two independent insertion events; the second event in the *N. tomentosiformis* lineage that gave rise to tobacco involved a recombination event between endogenous GRD, a free-living geminivirus, and potentially a mobile element, e.g., a helitron [74] (Figure 5).

GISH methods predominantly rely on dispersed repetitive sequences for differentiating subgenomes [75,76]. It had been the case that GISH to very small genomes resulted in no distinction or was limited to the few repeat-rich regions such as the pericentromeres and nucleolar organising regions; for a discussion see [8,77]. Ali *et al*. [78] showed that these problems could be overcome by increasing probe concentration and hybridisation time, facilitating the hybridisation of low-copy sequences. Using the new methodology, whole chromosome arms were painted, revealing the parental origin of *A. suecica* chromosomes** (AATT, 2*n* = 4*x* = 26), which is derived from *A. thaliana* (TT, 2*n* = 2*x* = 10) and *A. arenosa* (AAAA, 2*n* = 2*x* = 32) [78]. Sequences of the internal transcribed spacer (ITS) of rDNA indicate that *Arabidopsis suecica* has a single origin [79,80], probably about 12,000-300,000 years ago [81]. When GISH was used against a natural accession thought to be *A. suecica*, the plant was discovered to be a putative backcross of *A. suecica* to* A. arenosa*, with five *A. thaliana* and 24 *A. arenosa* chromosomes (AAAT, 2*n* = 29) [78]. Meiotic spreads showed that in one cell there were three unpaired *A. thaliana* chromosomes and two chromosomes paired allosyndetically (intergenomically), one in a bivalent, the other in a quadrivalent. Quadrivalent formation was attributed to close homeologue similarity as GISH was unable to resolve any intergenomic translocations [76]. Madlung *et al*. [82] showed in synthetic *A. suecica* (2*n* = 4*x* = 26) high levels (30%) of meiotic abnormalities, including bridges and chromosome fragments, but based on the centromeric signals using AaCEN and AtCEN probes, there was no evidence for allosyndetic pairing. Synthetic *A. suecica* also showed somatic instability, with aneuploid cells appearing to arise spontaneously and intermittently [83]. The stability of the natural species in comparison is presumably a result of the stabilising forces of selection in the wild.

Despite potential problems associated with multivalent formation in early allopolyploid divergence, the presence of some fixed translocations in allopolyploid species may suggest that some intergenomic exchanges are important in stabilising the genome [84,85] or alternatively may have arisen as a consequence of genetic drift. GISH detected large translocations in allopolyploids such as* Nicotiana tabacum* [86,87], *Avena sativa* [88,89], *Avena byzantina, Avena sterilis* [88],* Avena maroccana* [90], *Avena fatua* [91] and *Aegilops cylindrica* [92]. With *Avena sativa*,** GISH could not distinguish between A and D subgenomes, instead genome-specific painting was achieved using a dispersed repetitive sequence as a FISH probe [93].

In wheat polyploids, pairing control genes (PCGs) such as the *Ph1* locus greatly reduce homeologous pairing during meiosis [111,112]. Due to the presence of a functional *Ph1* locus, Mestiri *et al*. [113] did not observe allosyndetic pairing using GISH; however, the incidence of aneuploid plants was correlated with the frequency of univalents in the preceding meiosis. In *Ph1* mutants of *T. aestivum*, GISH showed that multivalents arise through both homeologous (see Figure 3) and homologous pairing of chromosomes [114]. Multivalents in the former case can result from reciprocal intergenomic translocations [114].

The stable integration of alien DNA following hybridisation with a diverged lineage, *i.e.*, introgression, is likely to represent an important process in plant evolution [94,95]. In most plants the occurrence or extent of introgression is not appreciated because of the detailed genomic studies that are required to confirm genetic exchange [96]. In nature, introgression is expected to be most prevalent where genetic divergence between species is lowest, as this is where the fewest barriers to gene flow are expected to have accumulated [97]. This is particularly so when the hybrid lineage is the same ploidy as the parents [54]. Introgression can be limited by genic incompatibilities or chromosomal incompatibilities resulting in infertility or hybrid inviability [98,99,100]. In natural diploid hybrids derived from *Helianthus annuus* and *H. petiolaris,* meiotic recombination** was reduced in regions containing inversions or translocations, whilst other recombinant genotypes had reduced pollen fertility [101]. It has been argued that by limiting recombination, changes in chromosome structure may promote speciation if they are close to regions which reduce fertility [102]. Chromosome doubling (polyploidy) before or after interspecific hybridisation may instantly generate isolation barriers because of the differences in ploidal levels. Chromosome doubling also creates identical homologous pairing partners in the hybrids, potentially leading to increased fertility [103,104,105].

## 4. Interspecific Hybridisation and Introgression in Plant Breeding

GISH has played an important role in integrating genetic and physical maps in cereal genetics [106,107]. Wheat (*Triticum aestivum*) is an allohexaploid derived from three ancestral diploid species (genome designation: AABBDD, 2*n* = 6*x* = 42) and is particularly amenable to alien chromatin introductions derived from complex interspecific hybrid combinations. Wheat addition lines of barley chromosome 5 [108], rye chromosome 1 [109] and barley chromosome 3 [110] were induced to undergo recombination between the alien and wheat chromosomes through the activities of the gametocidal chromosome (Gc) from *Aegilops cylindrica*. The recombinant products were then mapped by GISH, and PCR was used to confirm the genes carried by those segments. In this way it was possible to define the nature and distribution of the alien chromatin in the wheat lines as well as corroborate gene order from genetic maps.

The *Lolium-Festuca* complex includes natural hybrids and allopolyploids; there are also synthetic lines, which have been developed to improve fodder-crop quality. GISH can clearly distinguish the parental origin of the chromosomes and has been used to show that homeologous recombination is frequent in diploid and polyploid intergeneric hybrids, reviewed in [115]. Zwierzykowski [116] applied GISH to F_2_-F_6_ interbreeding allotetraploids generated from a cross between autotetraploids (2*n* = 4*x* = 28) of *Festuca pratensis* and *Lolium perenne*. Over five selfed generations, there was a trend towards an increasing number of recombinant chromosomes but also an increased representation of *Lolium* chromosomes based on centromeric GISH signals. The latter effect was explained by irregular meiotic configurations and centromeric drive, with chromosomes containing *Lolium* centromeres gradually substituting chromosomes with *Festuca* centromeres [116].

In tomato breeding programmes, GISH has been used to study the effect of different sized introgressed segments on meiotic pairing and recombination. Ji and Chetelat [117] painted segments of DNA introgressed from a wild relative (*Solanum lycopersicoides*) to homologues of chromosome 7. Previous work has shown that gene order on chromosome 7 is largely similar between species [118]. Longer alien introgressed segments reduced pairing and recombination between chromosome 7 homologues more than shorter segments [118].

Major structural changes such as translocations have also been shown in synthetic (oil seed rape) allopolyploids using genetic markers [119]. An examination of similar materials at meiosis revealed numerous aberrant meiotic divisions, e.g. multivalents, bridges and chromosomal laggards, and such processes provide a mechanism for the numerous structural changes observed after only a few generations [120]. Such changes frequently reduce fitness of the synthetic allopolyploids [119] and would be selected against in nature. Pairing control genes (PCGs), such as the wheat *Ph* genes, which ensure homologous pairing at meiosis, may have arisen many times in plant evolution, given that most established allopolyploids studied predominantly form bivalents [121]. Le Comber *et al.* [122] have suggested the PCGs may not be required to restore bivalent pairing; instead, processes such as gene subfunctionalisation and neofunctionalisation may accelerate subgenome divergence following allopolyploidisation.

## 5. Sequence Dynamics

Small-scale sequence changes are likely to be important in the homogenisation of subgenomes. Comparative genomic data have provided evidence of non-reciprocal recombination (e.g. gene conversion) of short DNA fragments [123], retrotransposition [124,125] and deletions [126] following hybridisation/allopolyploidisation. 

These changes can be rapid. In natural and synthetic allopolyploids of the Triticeae, deletions include the loss of genome- and chromosome-specific sequences within the first few generations [127,128,129,130], which leads to genome size reductions of approximately 1-2 Gbp [131]. Deletion of different repeat sequences was observed in the fourth generation of synthetic lines of *Nicotiana tabacum* [132]. These genetic changes, although widespread, may not necessarily result in gross karyotypic changes. In early-generation synthetic Triticeae allopolyploids, Han *et al.* [133] found no significant changes in chromosome structure with GISH, despite molecular analyses showing deletion or recombination among genic and retrotransposon sequences. In *Spartina*,** very few genetic alterations have been detected [134,135,136], and ISH data suggest that there has been little change in overall chromosome organisation [52]. However, sequence changes associated with transposable elements (TEs) have been detected and occur mostly as a result of hybridisation, rather than genome doubling [137].

Activation of transposable elements is a possible outcome of genome shock following hybridisation [138] and probably arises through alterations in epigenetically controlled TE suppression mechanisms [139]. In early generations of synthetic *Arabidopsis suecica* allopolyploids,* En-Spm* transposons and a *Copia* retrotransposon became transcriptionally active [82]. Likewise, early-generation synthetic Triticeae allopolyploids showed an increase in** transcripts** of the *Wis* retrotransposon, including chimeras originating from read-through into adjacent sequences [140]. In wide synthetic hybrids made from *Oryza sativa* and a wild relative (*Zizania latifolia*), *Gypsy* and *Copia* retrotransposons became transcriptionally active, and their genomic copy number increased; after a few generations, this activity was followed by strong transcriptional resuppression [141].

GISH studies in *Nicotiana* allopolyploids that arose at different times show a gradual reduction in GISH being able to differentiate subgenomes, rendering it ineffective in allopolyploids formed more than 5 million years ago (mya) [19,142]. The inferred loss of GISH signal over time is thought to be a consequence of ‘genome turnover’ processes [143] that include homogenisation, amplification and loss of repetitive DNA sequences such as retrotransposons [19,142]. Similar data were obtained from detailed analysis of BAC clones in rice, where it was estimated that there is nearly complete replacement of retroelements within 8 million years [48].

The distributions of *Tnt1* retrotransposons were mapped by FISH to the allotetraploid *N. tabacum* and its diploid progenitors *N. sylvestris* and *N. tomentosiformis* [144]. Whilst the distribution of elements broadly reflects the expectation in tobacco, there are also tobacco-specific signals, potentially reflecting insertions in tobacco or losses in the diploid parents since allopolyploidy. An examination of synthetic *N. tabacum* revealed evidence of significant amplification of young *Tnt1* elements in early generations, suggesting that hybridisation and polyploidy had released these elements from normal epigenetic controls [145]. Interestingly, it is the same family of elements (*Tnt1*) that also deviate from additivity in natural tobacco [144].

*Gossypium hirsutum* is an allotetraploid that formed 1-2 mya [146]. FISH to *G. hirsutum* using probes to six high-copy dispersed elements showed hybridisation to both subgenomes; but were absent in one diploid progenitor, *G. raimondii.* These data** indicate either mobility of the elements between subgenomes in *G. hirsutum* or perhaps a loss/divergence of the elements in *G. raimondii* [147]. Estimates of *Copia* retrotransposition rates in *G. hirsutum* suggest a gradual process, rather than an immediate burst following allopolyploidisation [148].

The centromeres of plants contain long arrays of one or several centromeric tandem repeats, with typically one or a few prominent repeat families being present on all chromosomes [149]. Studies of the two closest relatives of *Arabidopsis* *thaliana* and *A*. *arenosa* have highlighted the speed at which plant centromere repeats may be replaced by new variants. The centromeres of *Arabidopsis halleri* and *A. lyrata* contained two novel repeat families (pAge1, pAge2) as well as a previously described repeat (pAa, isolated from *Arabidopsis arenosa* [150]), but none of the repeats were detected on all chromosomes [151]. A detailed examination of centromere repeats among several accessions of *A. halleri* revealed varied distributions, suggesting transfer and/or rapid expansion and contraction of repeat variants [152]. A GISH study using genomic DNA from representatives of different tribes of the Poaceae to *Aegilops speltoides* chromosomes showed a cline of DNA conservation increasing from the pericentromeres to the core centromeric region [153]. A major component of the pericentromeres is retrotransposons, some of which appear to have increased in copy number relatively recently [154,155,156]. With FISH probes to centromere-specific retrotransposons, Liu *et al*. [157] could distinguish the subgenomes of Triticeae allopolyploids due to differences in element abundance and the resulting probe signal intensity.

## 6. Paleoallopolyploidy

There is growing evidence for paleopolyploidy in the ancestry of all angiosperms [158,159,160,161,162]. However, in older whole-genome duplications (WGDs), it is often unknown if they are the result of autopolyploidy or allopolyploidy. Confirming hybrid ancestry is confounded by the extensive changes in the genome as well as the divergence or extinction of parental lineages. Additional duplications through aneuploidy and segmental duplications of chromosomes add further complexity [163,164,165,166]. In the most recent WGD event of maize (*Zea mays* ssp*. mays*)** (2*n* = 20), which occurred approximately 11 mya, the genomes of two species were brought together [167]. Physical mapping suggests that the progenitors were both 2*n* = 20 [168], and a minimum of 17 fusions were responsible for the observed major rearrangements and reduction in chromosome number [164]. Since its most recent WGD, the maize genome has lost around 50% of its duplicated genes [126] and has become diploidised, forming 10 bivalents at meiosis [169]. Signatures of ancient allopolyploidisation in species of *Zea* can be uncovered by meiotic ISH analysis of synthetic hybrids. Triploids (3*n* = 30) derived from** maize and *Zea perennis* (2*n* = 20) with the constitution MMPPPP, typically show five bivalents (comprising PP chromosomes), trivalents (PPM) and univalents (M) [170]. The inclusion of five maize chromosomes in the trivalent suggests these chromosomes still harbour sequences with similarity to one of the ancestral genomes of *Z. perennis* [170].

FISH to centromeric regions has been used to provide evidence of ancient hybridisation events, where repeats appear to have remained distinct for several millions of years. This has been shown for soybean (2*n* = 2*x* = 40) through the isolation of novel centromeric repeats via genomic shotgun sequencing and screening for high-copy tandem repeats [171]. Two centromeric repeat sequences were identified, CentGM-1 and CentGM-2, which are 92 and 91 bp long, respectively. FISH showed that CentGM-1 and CentGM-2 exclusively hybridised to 12 and six pairs of chromosomes, respectively, with two other pairs of chromosomes carrying both repeats but in separate arrays [171]. The authors suggested that differential painting is due to hybridisation associated with the most recent WGD ~10-15 mya, based on the estimate by Schlueter *et al*. [172]. This conclusion has since been corroborated by a completed draft sequence of the soybean genome, with synteny revealing ancient homeologous blocks; researchers also estimate that the WGD took place ~13 mya [173].

Evidence for ancient hybridisation was provided for *Sorghum bicolor* (2*n* = 20). FISH using either a whole 45-kb centromeric BAC (22B2) or an independently derived clone (pSau3A10) containing 137 bp repeats strongly hybridised to 10 chromosomes and weakly to the other 10 [174,175]. Repeats similar to the 137-bp type also hybridised to some chromosomes of an interspecific hybrid of *Saccharum* [176]. If the pSau3A10 family of repeats did originate from the last known WGD, this implies that the repeat family has persisted for approximately 70 my since WGD in the common ancestor of the Poaceae [166,177]. Another centromeric repeat found in *S. bicolor*, pSau3A9, is also centromeric in several other grasses, including rice (subfamily Ehrhartoideae), maize (subfamily Panicoideae) and wheat (subfamily Pooideae) [178]. This repeat is distributed more uniformly amongst all the centromere regions of *S. bicolor* [178].

## 7. Next-Generation Sequencing (NGS), FISH Studies and the Future

FISH with markers that identify specific chromosomes provides a powerful approach to studying genomic change. However, there are few highly conserved sequences that can be used as probes across most plants, exceptions being 5S rDNA, 35S rDNA and telomere repeats. Genomic DNA cloned in high capacity vectors such as BACs has been used across species such as sorghum to maize [24] and *Arabidopsis thaliana* to different Brassicaceae species [31,33,34]. Outside of model plants and their close relatives, obtaining more FISH markers involves sequence isolation for each taxonomic group, requiring considerable, laborious work. Recent advances in NGS and bioinformatics now provide an alternative way to rapidly identify both dispersed and tandem repeat markers suitable for FISH at modest cost.

NGS enables large amounts of data to be generated rapidly from a complex mixture of genomic DNA. To identify repetitive sequences in the genome, an efficient approach is to carry out low-coverage (~1%) NGS, followed by a bioinformatics-based screening of repeats. Annotation can be aided by comparisons to known repeats present in databases of ESTs and TEs [179] or genomic sequences (NCBI; www.ncbi.nlm.nih.gov/). Non-coding simple sequence repeats can be detected based on search algorithms such as Tandem Repeat Finder [180]. Roche’s 454 sequencing is particularly useful if the genome under study is poorly characterised. This method generates copious amounts of sequence of an average length of 400 bp, sufficient to isolate and characterise dispersed and tandem repeats, even with low sequence coverage. When this approach is combined with FISH, it is possible to rapidly characterise genomes. Low-coverage genomic sequencing has been demonstrated for several plants. Swaminathan *et al.* [181] first used this approach for soybean, which has a haploid genome size of ~1.1 Gbp. Sequencing at ~7% coverage identified high-copy repeat families, such as telomeric and ribosomal DNA and also repeats with potential use as FISH probes, including TEs, centromeric and telomere-associated sequences.

Macas *et al*. [182] obtained repeats which together make up almost half of the pea genome, despite sequencing at less than 1% coverage. A subset of 14 repeats, estimated to be present at 2,000 to 51,000 copies per haploid genome, was localised by FISH. The distributions were predominantly centromeric or subtelomeric, on one to all seven pairs of chromosomes, with only one repeat being dispersed [182]. Wicker *et al.* [183] obtained ~1% coverage of the barley genome; total TE copy numbers inferred from this whole-genome sampling were compared to actual copy numbers in nine barley genomic BAC sequences. Discrepancies in TE abundance between the two datasets could be attributed to an unequal distribution in the genome, leading to an over- or underestimate based on the genomic position of the BACs. One example is the DNA transposon *Casper*, which was three-fold under-represented in the BAC sequences relative to its inferred genomic copy number; the transposon was localised mostly at the subtelomeres. We are currently using genomic 454 sequences to develop FISH markers for examining chromosomal changes in allotetraploid species of *Tragopogon*. Sequencing their respective diploid progenitors should not only provide many cytological markers, but also indicate those that are enriched in one of the parental genomes.

**Figure 6:**
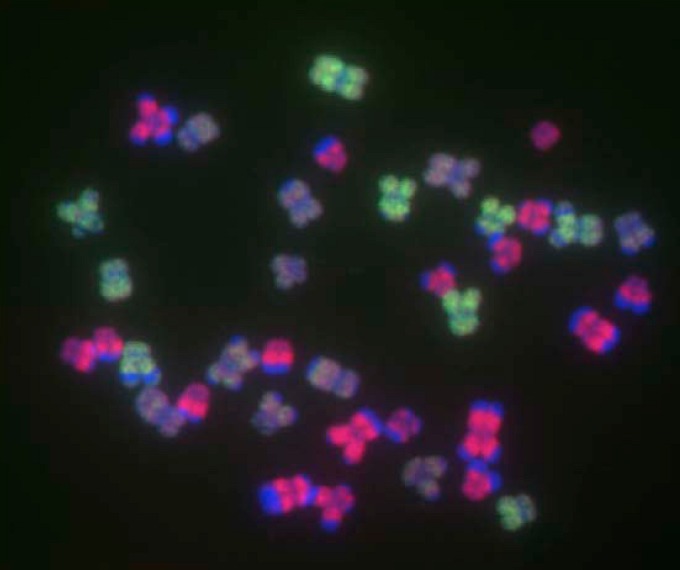
FISH to a partial root tip metaphase preparation of a *Tripsacum* x* Zea* trispecies hybrid (3*n =* 38). Chromosomes are hybridised by a mixture of genomic DNA clones, which have been directly labelled with fluorophores. Chromosomes can be identified as originating from *T. dactyloides* (red signal), *Z. mays* (green signal) or *Z. diploperennis* (mixed red and green signal); chromosomes are counterstained with DAPI (blue). Photo by Tatiana Arias, courtesy of James Birchler.

Another approach for developing non-dispersed FISH markers is to identify clusters of tandemly duplicated genes using a combination of ESTs, gene-mapping data and partial genome sequences (e.g. BACs). Kato *et al.* [184] applied 1.7 – 4 kb FISH probes to the tandemly repeated *rp1* and *rp3* resistance genes and the *α-zeinA* gene family, to paint single loci in the maize genome. Instead of using fluorescently labelled antibody for indirect detection, probes were directly labelled by a modified nick translation procedure that enables a high incorporation of dNTP-fluorophores [184]. Two advantages of this direct labelling method are that it reduces non-specific background, allowing smaller targets (~3 kb) to be detected, and it increases the number of probes that can be used simultaneously [184,185]. This method was used in a study of synthetic *Zea* ssp*.* x *Tripsacum dactyloides* hybrids and maize lines carrying introgressed DNA [186]. FISH with genome-enriched genomic clones, such as retrotransposon LTRs, were identified via Southern blotting and enabled the chromosomes of different species to be clearly distinguished. An example of the application of this method to a trispecies hybrid of *T.* *dactyloides* x *Z. mays* x *Z. diploperennis* (3*n =* 3*x* = 38) is shown in Figure 6.

One way of inferring changes that took place in early angiosperm evolution is to include representatives of the basal lineages, such as *Amborella trichopoda* (the sister to all other extant flowering plants) and the Nymphaeales (water lilies) reviewed in [187]. *Amborella* offers the unique ability to “root” analyses of all angiosperm features, from gene families to genome structure, and from physiology to morphology [188]** and has therefore been proposed as a candidate for complete nuclear genome sequencing, because it represents an evolutionary reference genome for all other angiosperms [188]. As part of this effort, FISH will be used to anchor BAC contigs to *Amborella* chromosomes and aid construction of a physical map.

## 8. Conclusions

Cytogenetics is poised to have an important role in plant biology into the future. The human population is expected to rise from 6 billion to 10 billion by 2100, resulting in huge increased demands on agriculture and land use. This problem is compounded by climate change [189], which has resulted in the growing of biofuel crops. Biofuels compete with land otherwise needed for food production or damages areas set aside for the conservation of biodiversity. Therefore, informed plant breeding to increase yield and quality and to improve farming practice is urgently needed. Modern cytogenetics will have a role in this, just as traditional cytogenetics was applied so successfully in the past. FISH will be used to map sequences and identify alien chromatin in new breeding lines. Another major issue facing humankind is conservation of biodiversity, an end to which most governments are committed, not least because it was estimated that ecosystem functioning was worth on average 33 trillion US dollars a year [190]. Characterizing that biodiversity is essential for conservation, and once again cytogenetics will have an important role in documenting biodiversity as well helping to reveal the processes that generate it. Cytogenetic methods in plant breeding and the study of biodiversity will therefore be required into the foreseeable future.
